# Electrospun Scaffolds as Antimicrobial Herbal Extract Delivery Vehicles for Wound Healing

**DOI:** 10.3390/jfb14090481

**Published:** 2023-09-19

**Authors:** Caglar Ersanli, Chrysoula (Chrysa) Voidarou, Athina Tzora, Konstantina Fotou, Dimitrios I. Zeugolis, Ioannis Skoufos

**Affiliations:** 1 Laboratory of Animal Science, Nutrition and Biotechnology, Department of Agriculture, University of Ioannina, 47100 Arta, Greece; c.ersanli@uoi.gr; 2 Laboratory of Animal Health, Food Hygiene and Quality, Department of Agriculture, University of Ioannina, 47100 Arta, Greecetzora@uoi.gr (A.T.); kfotou@uoi.gr (K.F.); 3 Regenerative, Modular & Developmental Engineering Laboratory (REMODEL), Charles Institute of Dermatology, Conway Institute of Biomolecular and Biomedical Research, School of Mechanical and Materials Engineering, University College Dublin, D04 V1W8 Dublin, Ireland; dimitrios.zevgolis@ucd.ie

**Keywords:** herbal extract, antibacterial, electrospinning, nanofibrous scaffold, delivery vehicle, wound healing

## Abstract

Herbal extracts have been used in traditional remedies since the earliest myths. They have excellent antimicrobial, anti-inflammatory, and antioxidant activities owing to various bioactive components in their structure. However, due to their inability to reach a target and low biostability, their use with a delivery vehicle has come into prominence. For this purpose, electrospun nanofibrous scaffolds have been widely preferred for the delivery and release of antimicrobial herbal extracts due to the flexibility and operational versatility of the electrospinning technique. Herein, we briefly reviewed the electrospun nanofibrous scaffolds as delivery systems for herbal extracts with a particular focus on the preclinical studies for wound-healing applications that have been published in the last five years. We also discussed the indirect effects of herbal extracts on wound healing by altering the characteristics of electrospun mats.

## 1. Introduction

A wound is the damage of a living tissue [1] which is caused intentionally (e.g., gunshots [2]) or unintentionally (e.g., skin cut, animal bite, traumas [3]) [4]. Although the human body has an excellent capability for healing wounds through a cascade of simultaneous phases, imperfect repair of damaged skin may cause vital damage, in particular the emergence of an infection, commonly in chronic wounds [5,6]. In contravention of the existence of various types of bacteria in the skin microbiota, biofilm formation or threshold value of substantial bacteria may block the wound-healing process [7]. The most common pathogenic strains that infect the wound site were revealed to be the *Staphylococcus aureus* (*S. aureus*), *Pseudomonas aeruginosa* (*P. aeruginosa*), and methicillin-resistant *S. aureus* [8]. Antibiotics are generally the first option for the treatment of infections, especially local ones [9]. However, overuse and maladministration of antibiotics [10,11,12] give rise to systemic toxicity and generation of antibiotic-resistant microorganisms. Therefore, due to the stated crucial concerns, alternative, unconventional, and non-antibiotic natural-based therapeutics (e.g., herbal extracts and essential oils) have come into prominence among most scientists as well as companies.

Herbal extracts have been used for traditional treatment purposes, e.g., burn wounds, bone fractures, and intestinal problems, since the earliest myths [13]. They demonstrate an excellent antimicrobial, antioxidant, anti-inflammatory, and anticarcinogenic activity owing to the bioactive compounds (e.g., polyphenols, vitamins) in their structure [14,15]. Almost 70% of people worldwide believe the primary health benefit of herbal compounds, according to the report by the World Health Organization [16]. Herbs present limitless sources to develop alternative, safe, and renewable therapeutics. For instance, among over two hundred and fifty thousand vascular plants, only around 17% of them have been researched for medicinal purposes [13]. Even though herbal extracts have been known for their excellent biological activities, some shortfalls still appear, such as poor biostability and the inability to reach the target [17]. Hence, there has been a need to develop engineered carrier and delivery vehicle systems (e.g., electrospun nanofibers, hydrogels) to increase the treatment and targeting efficacy.

**Figure 1:**
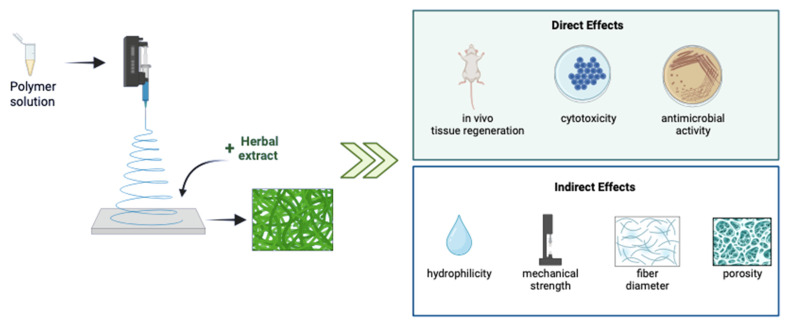
Direct and indirect effects of the incorporation of herbal extracts into electrospun biomaterials. This figure was created using BioRender.com.

Electrospinning is a simple and effective technique to fabricate various size ranges of nanofibers using electric force to pull charged threads of polymer solutions either with or without including any herbal extract [18]. Moreover, electrospinning can provide the sustained and targeted release of therapeutics [19]. One of the promising advantages of this technique is its operational versatility and flexibility to achieve desirable surface characteristics like large surface-to-volume ratio, and desired porosity [20,21]. It is a favorable method for the spinning of several kinds of materials such as natural, synthetic, or blended polymers. Nevertheless, several health concerns related to the usage of synthetic materials started to appear [18]. Consequently, the perspective shifted from a preference for synthetic to one for natural materials. In this manner, herbal extracts have become rising stars as attractive sources of electrospun biomaterials for many applications [22]. Herein, we briefly describe the role of antimicrobial herbal extracts incorporated in electrospun scaffold delivery systems (Figure 1), with a particular focus on preclinical studies for wound-healing applications that have been published in the last five years.

## 2. Direct Effect of Herbal Extracts on Wound Healing

Herbal extracts are outstanding and alternative antimicrobial compounds, as opposed to common, traditional therapeutic agents (e.g., antibiotics), owing to their excellent biological properties, such as antimicrobial, antioxidant, anticarcinogenic, and anti-inflammatory activities, arising from various bioactive phytochemicals in their structure. Herbal extracts can regenerate damaged tissue in the wound area and fasten the healing process thanks to these bioactive components [23,24]. Furthermore, these phytochemicals can exert their antimicrobial action by damaging the bacterial cell wall and membrane, blocking or disrupting the synthesis of crucial bacterial proteins, as well as inhibiting the action of significant bacterial metabolic pathways (e.g., DNA replication) [15,25,26]. The incorporation of herbal extracts into electrospun nanofibrous mats has been extensively studied to enhance their biostability, despite their effective bioactivity.

The preclinical studies revealed that the treatment of wounds with herbal extract including nanofibers enhances the in vivo wound-healing rate (Table 1). The 20% (*w/w*) of *Malva sylvestris* extract incorporated in a polyurethane/carboxymethyl cellulose (PU/CMC) mats yielded a complete wound closure on the 14th post-treatment day, whilst its lower concentrations (5–10% *w/w*) were not sufficient. Due to the presence of various polysaccharides (e.g., flavonoids, naphthoquinones, and anthocyanins), *M. sylvestris* displayed good biological activity in a full-thickness diabetic wound model [27]. The addition of *Calendula officinalis* increased the closure rate of a wound treated with a pristine chitosan/polyethylene oxide (Chi/PEO) nanofibrous mat for 14 days from 80% to 90% [28]. In a study, peppermint extract and gelatin nanoparticles (NPs) were embedded into a PU/Pluronic F127 nanofibrous membrane to enhance wound healing. While both extract- and gelatin NPs-loaded membranes showed a 97% wound recovery rate, the scaffolds that contained only peppermint extract presented an almost 75% wound-closure rate within 21 days [29]. The treatment of wounds on a BALB/c mice model with henna extract-loaded gelatin/oxidized starch nanofibers presented a reduction in the number of macrophages, an inflammatory response with a thinner inflammation zone, and enhanced collagen deposition, which might have been due to the existence of various aromatic hydrocarbons in the henna structure [30]. The addition of palmatine accelerated the wound-healing ability of the PCL/gelatin nanofibers on the rabbit ear model by decreasing the healing time from 14 to 12 days, as well as inhibited hypertrophic scar formation. However, the highest concentration of palmatine (8 *w*%) showed cytotoxicity on the L929 fibroblasts [31]. In addition, almost 97% of the wound on the Wistar rat recovered due to the synergetic effect of the *Achyranthes aspera* and *Datura metel* extracts, which were incorporated into the PCL nanofibrous mats within 9 days post-treatment [32].

According to Table 1, when compared to extracts which were applied in the same concentration units (%, *w/w*), the addition of 2% of *Lawsonia inermis* extract [33] led to the almost complete wound closure at day 14, whilst the 15% of *Malva sylvestris* [27], 1% of curcumin [34], and 15% of peppermint [29] extracts displayed around 95%, 75%, and 65% in vivo wound-closure rates, respectively. Moreover, 2–8% of palmatine addition into the PCL/gelatin nanofibers [31] yielded an almost complete wound closure in 12 days. Additionally, none of the extracts showed any cytotoxic effect. Hence, it may be concluded that palmatine is the most effective extract with its lower concentrations for wound healing, followed by the *Lawsonia inermis*, *Malva sylvestris*, curcumin, and peppermint extracts.

**Table 1:** Indicative examples of electrospun scaffolds as antimicrobial herbal extract delivery vehicles for wound-healing applications published in the last five years. Abbreviations: antimicrobial susceptibility test: AST; Chitosan: Chi; Polycaprolactone: PCL; Poly (lactic acid): PLA; Poly(vinyl alcohol): PVA; Carboxymethyl cellulose: CMC; Polyurethane: PU; Poly(ethylene glycol): PEG; Nanoparticle: NP; Poly(lactic-co-glycolic acid): PLGA; Collagen: Col; Carboxyethyl chitosan: CE-Chi; Poly(ethylene oxide): PEO; Sodium tripolyphosphate: TPP; Microparticle: MP; Poly(hydroxy butyrate): PHB; Graphene oxide: GO; Poly-L-lactic acid: PLLA; PCL-PEG–block copolymer: PCL-b-PEG; Poly(3-hydroxybutyrate-co-3-hydroxyvalerate): PHBV; Polyvinyl pyrrolidone: PVP; (2,2,6,6-Tetramethylpiperidin-1-yl)oxyl: TEMPO; TEMPO oxidized cellulose nanofiber: TOCN; Polyethylene glycol methyl ether methacrylate: PEGMA; Gulmohar seed polysaccharide: GSP; Glutaraldehyde: GTA; human mesenchymal stem cell: hMSC; human dermal fibroblast: HDF; human keratinocytes cell line: HaCaT; human umbilical cord matrix: hUCM; normal human foreskin: NHF; human umbilical vein endothelial cell: HUVEC; *Propionibacterium acnes*: *P. acnes*; *Corynebacterium diphtheriae*: *C. diphtheriae*; *Staphylococcus epidermidis*: *S. epidermidis*; *Lactobacillus acidophilus*: *L. acidophilus*; *Bacillus subtilis*: *B. subtilis*; *Escherichia coli*: *E. coli*; *Vibrio parahaemolyticus*: *V. parahaemolyticus*; *Pseudomonas otitidis*: *P. otitidis*; *Klebsiella pneumoniae*: *K. pneumoniae*; *Staphylococcus aureus*: *S. aureus*; Methicillin-resistant *Staphylococcus aureus*: MRSA; *Pseudomonas aeruginosa*: *P. aeruginosa*.

Scaffold Conformation	Herbal Additive	AST	Antibacterial Activity Against	Cell Line	In Vivo Model	Important Biological Activity Outcome	Refs.
cellulose acetate (5 g) nanofibrous mat No crosslink	annatto extract (20 mL)	-	-	mouse fibroblasts	Wistar rat wound model	Annatto extract modulated the inflammation process.	[35]
PVA (10% *w/v*)/Chi (3% *w/v*) nanofibrous mat No crosslink	spray-dried *Centella asiatica*, *Portulaca oleracea*, and *Houttuynia cordata* extracts (3, 6, and 9% *w/w* of each)	disc diffusion	*P. acnes*	Chick chorioallantoic membrane (CAM) in vitro model	10 patients with mild-to-moderate facial *Acne vulgaris* aged between 20 and 30	Developed patches presented good bacterial inhibition for *P. acnes*, while they were not effective for pathogenic *E. coli* and *S. aureus*.	[36]
PCL (14% *w/v*) nanofibrous mat No crosslink	*Achyranthes aspera* and *Datura metel* leaf extracts (10, 20, and 30% *w/v*)	well diffusion	*C. diphtheriae*, *Enterococcus* spp., *S. epidermidis*, *L. acidophilus*, *B. subtilis*, *E. coli*, *Shigella* spp., *V. parahaemolyticu*, *Pseudomonas* spp., *P. otitidis*, *K. pneumoniae*, and *Vibrio* spp.	the vero kidney cells	male Wistar rat wound model	Hybrid scaffolds recovered the wound in vivo within 9 days.	[32]
PU/CMC (20% *w/w*) nanofibrous mat (various PU:CMC *w* ratio) No crosslink	*Malva sylvestris* dried leaf extract (5–20% *w/w*)	agar dilution	*S. aureus*, and *E. coli*	hMSCs	male Wistar rat full-thickness diabetic wound model	Developed dressings containing 15% *w*/*w* extract showed about 95% of wound-healing rate within day 14.	[27]
Chi (2% *w/v*)/PEO (3% *w/v*) nanofibrous mat crosslinked with 25% *v/v* GTA vapor	*Calendula officinalis* (1, 2, and 3% *w/v*)	agar well diffusion, viable cell count method	*S. aureus*, and *E. coli*	HDFs	male Wistar rat full-thickness wound model	Developed hybrid scaffold led to 87.5% of wound closure after 14 days.	[28]
PVA (10 *w*%)/guar gum (1 *w*%) nanofibrous mat crosslinked with 5 *w*% citric acid	*Acalypha indica*, *Aristolochia bracteolate*, *Thespesia populnea*, and *Lawsonia inermis* (henna) extracts (20 *w*% of total polymer weight)	-	-	MSCs	female Wistar rat splint excisional model	In vivo efficacy of the hybrid scaffold with/without MSCs showed complete wound restoration with minimal scarring.	[37]
PU (7% *w/v*)/pluronic F127 (0.7% *w/v*) nanofibrous mat crosslinked with 5–20% *w/w* gelatin NPs	peppermint ethanolic leaf extract (15% *w/w*)	agar dilution	*S. aureus*, and *E. coli*	hUCM cells	male Wistar rat diabetic wound model	The addition of 15% extract and 10% NPs as a crosslinker enhanced the wound-closure rate from 75% to 95% within 21 days.	[29]
Gelatin (13 *w*%)/oxidized starch (5% *w/v*) nanofibrous mat crosslinked with 2% *w/v* oxidized starch	*Lawsonia inermis* (henna) aqueous leaf extract (10–40 *v*%)	disc diffusion, liquid medium microdilution	*S. aureus*, and *E. coli*	L929 fibroblasts	BALB/c mice second-degree burn wound model	The implantation of wounds treated with 30% henna-loaded mats exhibited clear epithelialization, angiogenesis, well-organized collagen molecules, and hair follicles on the fourth post-treatment day.	[30]
PVA (10–30% *w/v*) nanofibrous mat No crosslink	propolis dried alcoholic extract (1.25 mg/mL)	-	-	NIH 3T3 fibroblasts	male Swiss mice diabetic wound model	Propolis-loaded dressings showed partial wound closure (68%) within 7 days.	[38]
PCL (20% *w/v*)/gelatin (8% *w/v*) nanofibrous mat No crosslink	palmatine (2, 5, and 8 *w*%)	disc diffusion	*S. aureus*, and *E. coli*	L929 fibroblasts	rabbit ear model of hypertrophic scar (HS)	Sustained release of palmatine led to inhibition of HS formation, as well as accelerated wound healing.	[31]
PCL (10% *w/v*)/Mesoporous silica (5 *w*%) nanofibrous mat No crosslink	curcumin (1 *w*%)	well diffusion	*S. aureus*, and *E. coli*	3T6 Swiss cells	female Wistar albino rat full-thickness excision skin wound model	The incorporation of both curcumin and silica contributed to 99% of scarless wound healing in vivo within 21 days.	[34]
Gelatin (17 *w*%)/PVA (10 *w*%)/Chi (2 *w*%) bilayer nanofibrous mat crosslinked with 50 *w*% GTA vapors for 45 min.	curcumin (400 µg/mL), and *Lithospermi radix* extract (625 µg/mL)	-	-	L929 fibroblasts	male SD rat streptozotocin-induced diabetic wound model	While single incorporation of curcumin and LR extract increased the TGF-ß secretion level and collagen synthesis, respectively, their synergetic effect demonstrated curative activity.	[39]
Chi (3 *w*%)/PEO (4 *w*%) nanofibrous mat No crosslink	*Lawsonia inermis* (henna) and ethanolic leaf extract (1, and 2 *w*%)	disc diffusion	*S. aureus*, and *E. coli*	NHF fibroblasts	male Wistar rat wound model	The synergetic effect of henna extract and Chi polymer promoted antibacterial activity, biocompatibility, and wound-healing rate.	[33]
Chi (3 *w*%)/PEO (3 *w*%) nanofibrous mat No crosslink	*Aloe vera*	agar well diffusion	*S. aureus*, and *E. coli*	NIH 3T3 fibroblasts	Swiss albino mice wound model	*Aloe vera*-incorporated mats gave better in vitro and in vivo results when compared to pristine mats.	[40]
PCL (8 *w*%)/Gelatin (4, 8 *w*%) core/shell nanofibrous mat No crosslink	*Gymnema sylvestre* ultrasound-assisted and cold-macerated leaf extracts (10 *w*%) with minocycline hydrochloride (2 *w*%)	disc diffusion, bacterial cell viability assay	*S. aureus*, MRSA, *S. epidermidis*, *P. aeruginosa*, and *E. coli*	HDFs, and HaCaTs	female porcine second-degree burn wound model	Developed composite mats showed a potent bactericidal effect against biofilm-forming pathogenic bacterial strains which can prolong wound healing.	[41]
PCL (4% *w/v*)/Gelatin (10% *w/v*) nanofibrous mat No crosslink	Lawsone (0.5, 1, and 1.5%)	disc diffusion	*S. aureus*	human normal gingival fibroblasts	male Wistar rat excision wound model	The expression of healing-related genes TGF-B1 and COL1 significantly increased on extract-loaded mats.	[42]
PCL (10% *w/v*) nanofibrous mat No crosslink	*Areca catechu* petroleum ether phytoextracts (5% *w/w*)	disc diffusion	*S. aureus*, and *P. aeruginosa*	L929 fibroblasts	female SD rat skin wound model	Phytoextracts showed slightly higher antibacterial activity on gram-positive *S. aureus* when compared to gram-negative *P. aeruginosa*.	[43]
PVA (6% *w/v*) nanofibrous mat crosslinked with 10% *v/v* GTA vapor for 10 h at ambient temperature	*Cordia myxa* ethanolic fruit extract (2.5, and 5% *w/v*)	-	-	human foreskin fibroblasts	male albino mice skin wound model	The 5% *w/v* extract addition to mats supported fibroblast proliferation and attachment while being non-cytotoxic to the cells.	[44]
Col (10, 11 *w*%)/PCL (10, 11 *w*%) three-layered nanofibrous mat No crosslink	*Melilotus officinalis* (2, 4, and 8% *w/w* based on collagen weight)	-	-	L929 fibroblasts	male Wistar rat diabetic wound model	The 8% *w/w* extract-added mats demonstrated favorable reepithelization of in vivo diabetic wounds as well collagen production and deposition.	[45]
Gelatin (10% *w/v*)/PCL (10% *w/v*)/PU (10% *w/v*) membrane/nanofiber bilayer scaffold No crosslink	propolis ethanolic extract (0.5% *w/w*)	disc diffusion	*S. aureus*, *S. epidermidis*, and *E. coli*	L929 fibroblasts	female Wistar rat skin wound model	Approximately 40% of the loaded extract was released within 50 h, which showed significant antibacterial activity against *S. aureus*.	[46]
PLGA (12% *w/v*)/PVA (0.5% *w/v*) nanofibrous mat No crosslink	*Aloe vera* (250 mg/mL) caged into lipid NPs (25 mg/mL)	-	-	HaCaTs, and BalbC/3T3 A31 fibroblasts	male *db/db* mice full-thickness wound model	The extract-caged lipid NPs directly loaded into mats did not show a difference in wound closure and reepithelization rates, as well as cell adhesion percentage.	[47]
PU (10% *w/v*) nanofibrous mat No crosslink	*Nigella sativa* oil (10% *v/v*)	ASTM E2149, and shake flask test	*S. aureus*, and *E. coli*	HUVECs	female SD rat	The synergetic effect of the nanofibers’ advantage and bioactive oil presented the fastest wound closure in vivo.	[48]

## 3. Indirect Effect of Herbal Extracts on Wound Healing

In addition to their direct effect on wound healing due to their bioactive compounds, herbal extracts may enhance the wound healing ability of electrospun nanofibers by altering their physicochemical, mechanical, and morphological characteristics. At this point, we will review how herbal extracts affected the hydrophilicity, mechanical strength, average fiber diameter, and porosity of electrospun nanofibrous wound dressings that provide benefits such as improved wound-healing.

### 3.1. Effect of Herbal Extracts on the Hydrophilicity of Electrospun Mats

The water uptake capacity of electrospun mats plays a crucial role in wound healing. The fabricated mats can conserve the moisture and nutrients in the wound area, as well as promote cell adhesion and proliferation with a higher swelling ratio [42,49]. The water uptake percentage of the Chi/PEO nanofibers increased from 93% to 119% through the incorporation of 2 *w%* of hydro-alcoholic henna extract, which was attributed to the hydrophilic functional groups of henna, while wound-closure rates were evaluated to be approximately 85% and 90% for pristine and 2 *w%* henna-including mats, respectively [33]. Similarly, the addition of lawsone raised the water content of the PCL/gelatin nanofibers by almost three-fold in the PBS (pH:7) media. This composite mat displayed a lower number of inflammatory cells and more organized fibroblasts with accelerated wound-healing within 14 days [42]. Additionally, enhanced wettability of nanofibrous mats may benefit the diffusion of nutrients to the wound area, absorption of exudate, as well enhance cell binding. For example, the presence of several polar phytochemicals in the *Gymnema sylvestre* extract achieved a decrease in the water contact angle of the PCL/gelatin nanofibers and a slight increase in the wound recovery percentage on the 32nd day post-injury [42].

### 3.2. Effect of Herbal Extracts on the Mechanical Strength of Electrospun Mats

The adequate mechanical properties of engineered electrospun mats are another important parameter to promote the formation of new dermal tissue and resist biodegradation during the wound-healing process [50]. An ideal nanofibrous wound dressing should provide a balance between flexibility and hardness [51] and display a tensile strength in the range of 0.8 to 18 MPa, which is proper for dermal cell culture and skin tissue engineering applications [52,53]. Herbal extracts may act as a reinforcement agent [37,41] and increase the tensile strength of nanofibers, which leads to the enhancement of wound recovery. To exemplify, the incorporation of *Acalypha indica*, *Aristolochia bracteolate*, *Thespesia populnea*, and henna extracts together increased the tensile strength of the PVA/Guar gum nanofibers because of the crosslinking impact of *A. bracteolate* and *A. indica* extracts resulting from their increased nonpolar functional ratio. Although the improved mechanical properties of mats are not the only effective parameter, polyherbal extract-including mats showed a slightly higher wound-closure rate (97%) than pristine mats (93%) within 14 days [37]. A similar effect was observed with the addition of the *Gymnema sylvestre* extract, which improved the ultimate tensile stress of the PCL/gelatin mats from about 1.4 to 4.3 MPa, an effect which may be attributed to the high number of hydrogen-bonding donor molecules in the structure of the extract. In parallel, the presence of an extract in the core/shell nanofibers, which are one of the most common nanofiber structures, significantly improved wound-closure percentage with enhanced epidermal cell proliferation [41]. However, in some studies, herbal extracts showed a plasticizing effect [20,28,33,42], resulting in the reduction of the tensile strength. Nevertheless, despite the decreased mechanical strength, the wound-healing ability of mats was not negatively affected.

### 3.3. Effect of Herbal Extracts on the Average Fiber Diameter of Electrospun Mats

The morphology of nanofibrous dressings plays a significant role in wound-healing applications, since the random orientation of nanofibers can mimic the nature of the extracellular matrix (ECM) [54]. As a general trend, the incorporation of herbal extract reduced the viscosity of the polymeric spinning solution with the increase in the conductivity, which induces the formation of smaller fibers since it acts as a plasticizer when added to a polymer blend [28,35,36,39,44,48]. Smaller-diameter nanofibers have a supportive effect on the wound-healing process due to their greater protein-absorption capacity. In other words, cell adhesion on smaller-diameter fibers is promoted due to their larger specific surface areas [55]. To illustrate, while the average diameter of the gelatin/PVA/Chi nanofibers was reduced almost two-fold by the incorporation of 400 µg/mL of curcumin, the curcumin-including composite dressings displayed greater wound recovery on the 14th day of treatment when compared to gauze control [39]. In contrast, it was reported that the addition of the palmatine [31] and *Melilotus officinalis* [45] extracts raised the average nanofiber diameters, which might be explained by the decreased electrical conductivity of the polymeric spinning solution. Nonetheless, the higher nanofiber diameters did not show an inhibitory effect on wound healing; even the acceleration of wound-healing rates [31] and more collagen deposition [45] were revealed. In summary, it can be concluded that, even though the general view is that of decreasing the nanofiber diameter by adding an herbal extract into the formulation, some studies showed opposite outcomes.

### 3.4. Effect of Herbal Extracts on the Porosity of Electrospun Mats

Besides the nanofiber dimension, porosity is accepted as another outstanding parameter for fibrous scaffolds [56]. Since the proper porosity allows the permeation of oxygen through the wound bed, it can benefit the acceleration of wound healing [57] by improving the proliferation of fibroblasts and keratinocytes. The resulting proliferating environment can lead to the reepithelization and formation of granulation tissue, advancing the secretion of wound-healing mediators (e.g., angiogenic factors, growth factors, and collagen) [47,54]. Case in point, the addition of 5% (*w/v*) of *Cordia myxa* ethanolic fruit extract increased the porosity of the PVA nanofibers by 11.8%, as well as provided the proper reepithelization, more collagen deposition, and a 33.6% smaller wound within 14 days [44]. Similarly, the incorporation of *Aloe vera* [56] and *Nigella sativa* oil [48] enhanced the porosity of the PLGA/PVA and PU nanofibrous scaffolds, respectively, with improved proliferation and wound-healing activity. The increased porosity might be explained by the thinner nanofibers produced through the addition of herbal extracts. Instead, the presence of various concentrations of *Calendula officinalis* did not affect the porosity of the Chi/PEO nanofibers [28].

## 4. Future Aspects

Electrospinning is one of the biomaterial fabrication processes that use the high-voltage-electric field to draw charged polymer melts/solutions through the collector to obtain nano-sized structures. Electrospinning has come into prominence when compared to traditional fabrication methods since it is a simple and user-friendly process that leads to control over the porosity and/or morphology of nanofibers by altering the fabrication parameters (e.g., flowrate, voltage of electric fields, and nozzle diameter). Moreover, electrospun nanofibers display various advantages for tissue engineering applications thanks to the possibility of adjusting the hydrophilicity and stimuli-responsive capacity of the fabricated materials. In particular, electrospun nanofibers are an excellent candidate for a wound dressing due to their high surface area to volume ratio, adjustable and high porosity, good biocompatibility, and mechanical properties. All these outstanding features favor cell attachment, growth, and proliferation, as well as wound moisturizing. In addition, due to growing health concerns regarding the use of synthetic molecules, nature-inspired molecules such as herbal extracts are constantly attracting attention for advancing greener and non-toxic products for wound-healing treatment. Therefore, we believe that electrospinning is a promising technology for developing wound dressings that incorporate natural therapeutics in the formulation of the nanofibers.

## 5. Conclusions

Wound care is a problem that has always concerned human health, from the beginning of humanity. In the quest for the treatment of infected wounds, herbal extracts have been slighted when compared to antibiotics, despite the fact that they have been a primary source of traditional remedies since ancient times. However, in the perspective of the development of alternative wound dressings, the study of herbal extract-incorporated delivery systems has recently begun to gain an important place in the literature to overcome the drawbacks of antibiotic usage, e.g., antimicrobial resistance and biofilm formation. Furthermore, electrospun scaffolds are one of the most prominent biomaterial forms owing to their highly porous, nano-sized structures. Through the incorporation of herbal extracts instead of synthetics, the reduction of several health concerns caused by the use of synthetics has been achieved. This review clearly exemplifies both the direct and indirect effects of herbal extracts on wound healing and gives countenance to the advancing of natural, herbal-based nanofibrous delivery systems for effective wound care and infectious treatment.

## Data Availability

Data are contained within the article.
